# A case report of intracholecystic papillary neoplasm of the gallbladder resembling a submucosal tumor

**DOI:** 10.1186/s40792-018-0524-2

**Published:** 2018-09-27

**Authors:** Ryo Muranushi, Hideyuki Saito, Asuka Matsumoto, Toshihide Kato, Naritaka Tanaka, Kenji Nakazato, Nobuhiro Morinaga, Yoshinori Shitara, Masatoshi Ishizaki, Takatomo Yoshida, Shinichi Aishima, Ken Shirabe

**Affiliations:** 1 Department of Hepatobiliary and Pancreatic Surgery, Gunma University Graduate School of Medicine, Gunma University, 3-39-15 Showa-Machi, Maebashi, Gunma 371-8511 Japan; 2 Department of Surgery, Fujioka General Hospital, Fujioka, Gunma Japan; 3 Department of Pathology, Fujioka General Hospital, Fujioka, Gunma Japan; 4 Department of Pathology and Microbiology, Saga University, Saga, Japan

**Keywords:** Intracholecystic papillary neoplasm, Adenomyomatous hyperplasia, Laparoscopic cholecystectomy

## Abstract

**Background:**

Intracholecystic papillary neoplasm (ICPN) is defined as papillary tumors detected macroscopically in the gallbladder. We report a case of ICPN which exhibited the atypical form like a submucosal tumor.

**Case presentation:**

A 70-year-old man was admitted to our hospital because of hepatic disorder. Computed tomography and magnetic resonance imaging showed irregular thickening of the wall within the gallbladder fundus. Because the lesion might have been malignant, we performed laparoscopic cholecystectomy and liver bed resection. Macroscopic findings showed the mucosal surface of the tumor was smooth, and its form was similar to that of a submucosal tumor. Histopathological examination revealed papillary tumors within the mass with low-grade dysplasia; therefore, we diagnosed ICPN.

**Conclusion:**

In the present case, ICPN was resembling a submucosal tumor macroscopically because the tumors arose into the Rokitansky-Aschoff sinus and the adenomyomatous hyperplasia was merged with the ICPN. It is necessary to consider the possibility of tumor lesions within adenomyomatous hyperplasia.

## Background

Intracholecystic papillary neoplasm (ICPN) is preinvasive neoplastic lesions characterized by papillary growth in the gallbladder. ICPN is defined as gallbladder lesions of intraductal papillary neoplasm of the bile duct (IPNB). IPNB is a premalignant lesion of the biliary tract and is counterpart of intraductal papillary-mucinous neoplasm (IPMN) in the pancreatic duct epithelium [1]. ICPN is a papillary tumor generally detected macroscopically and is sometimes diagnosed by imaging findings. Herein, we report a case of ICPN which exhibited atypical form and which was distinguished difficultly from gallbladder adenocarcinoma.

Through this case, we consider clinicopathological characteristics and therapeutic strategies of ICPN.

## Case presentation

**Fig. 1:**
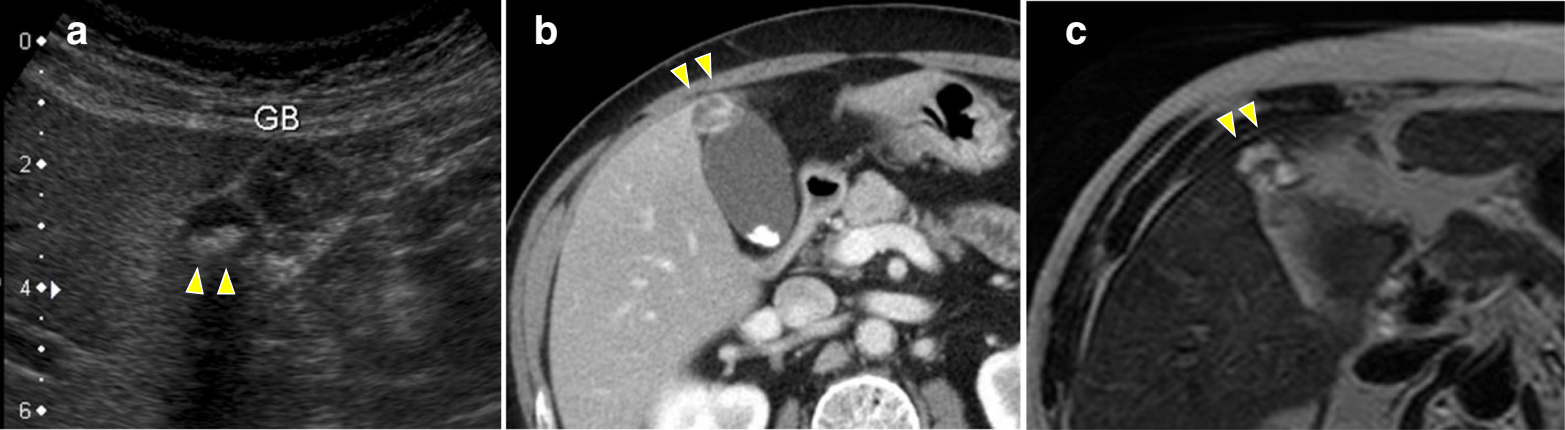
Preoperative imaging findings. **a** Abdominal ultrasonography showed an 8 × 7-mm solid mass at the gallbladder fundus. **b** Enhanced CT showed that irregular wall thickening at the gallbladder fundus, and the boundary between the tumor and liver was indistinct. **c** T2-weighted MRI showed high-intensity nodules inside the thickened wall of the gallbladder

**Fig. 2:**
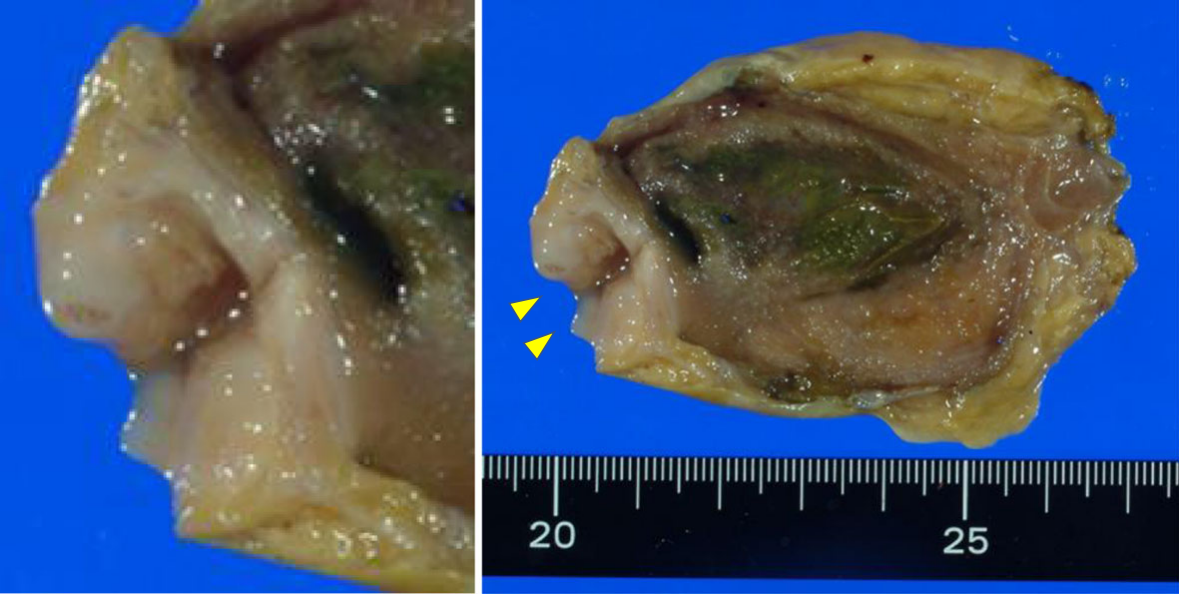
Photograph of resected specimen. It shows the gallbladder fundus on the left side and the cystic duct on the right side. A 15 × 10-mm mass like a submucosal tumor is visible within the gallbladder fundus (arrow), and its cut surface shows the papillary lesions

**Fig. 3:**
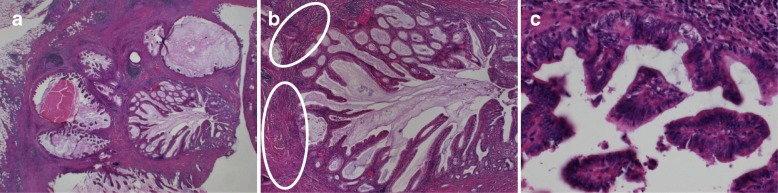
Histopathological findings. **a** Papillary tumors with cyst formation which presented mucin secretion were detected, and the Rokitansky-Aschoff sinus was formed (hematoxylin-eosin staining, × 40). **b** The smooth muscle became hyperplastic in the stromal tissue surrounding the papillary tumors (circle) (hematoxylin-eosin staining, × 100). **b** Epithelial cells. The nucleus chromatin increased, the karyotype was irregular, and the nuclear body became clear, showing low-grade dysplasia. (Hematoxylin-eosin staining, × 400)

**Fig. 4:**
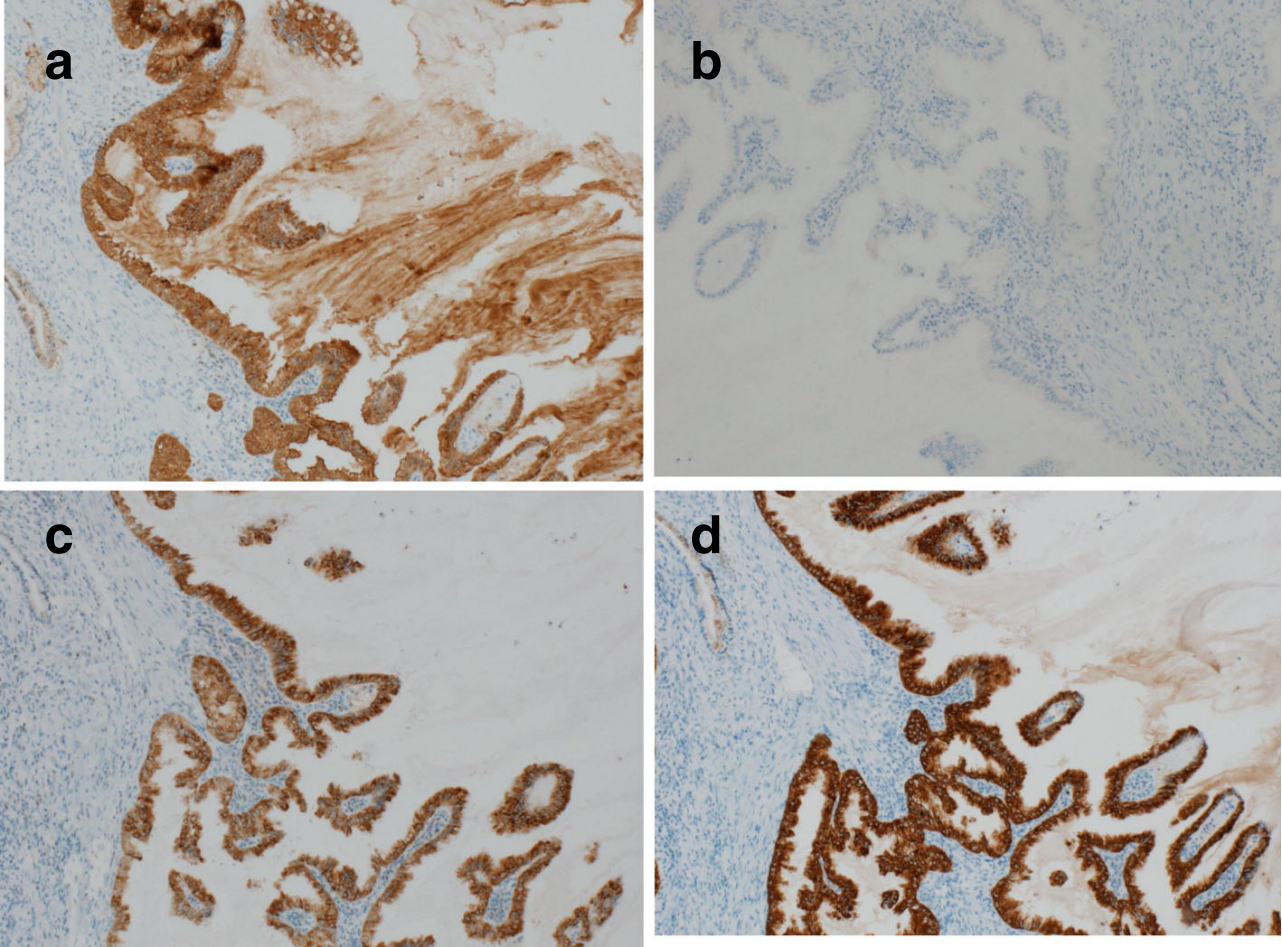
Immunohistochemical analysis of mucosal characteristics. **a** MUC1 was positive; **b** MUC2 was negative; **c** MUC5AC was positive; and **d** MUC6 was positive

The patient was a 70-year-old man. He was admitted to our hospital because of a hepatic disorder that was discovered during a routine health examination. Blood tests showed aspartate aminotransferase 48 U/L (normal range, 13 to 33 U/L), alanine phosphatase 66 U/L (normal range, 8.0 to 42 U/L), alkaline phosphatase 263 U/L (normal range, 115 to 359 U/L), gamma-glutamyl transpeptidase 100 (normal range, 10 to 47 IU/L), total bilirubin 0.5 mg/dL (normal range, 0.2 to 1.2 mg/dL), carcinoembryonic antigen 4.4 ng/mL (normal range, < 5.0 ng/ml), and carbohydrate antigen 19-9 10.4 U/mL (normal range, < 15 U/mL). Abdominal ultrasonography showed an 8 × 7-mm solid mass at the gallbladder fundus and several stones in the gallbladder (Fig. 1a). Enhanced computed tomography (CT) showed that irregular wall thickening at the gallbladder fundus and the boundary between tumor and the liver was indistinct (Fig. 1b). T2-weighted magnetic resonance imaging (MRI) showed a high-intensity nodule inside the thickened wall at the gallbladder fundus (Fig. 1c). According to these findings, we diagnosed the lesion as suspicious of malignancy and decided to perform surgery. During surgery, a tumor of approximately10 mm was found at the gallbladder fundus and color change of the liver bed floor adjacent to the tumor was detected. We performed laparoscopic cholecystectomy and liver bed resection. The macroscopic findings of the resected specimen showed a 15 × 10-mm milky yellow mass at the gallbladder fundus, and its cut surface showed papillary lesions (Fig. 2). The tumor mucosal surface was smooth, and its form was similar to that of a submucosal tumor. Histopathological findings showed papillary tumors with cyst formation, and the tumors represented mucin secretion (Fig. 3a). Additionally, the Rokitansky-Aschoff sinus (RAS) was formed, and the smooth muscle became hyperplasia in the stromal tissue surrounding the papillary tumors (Fig. 3a, b). Within the epithelial cells, nucleus chromatin increased, karyotype was irregular, and nuclear body became clear. There was no invasion into the stromal tissue. These findings demonstrated ICPN with low-grade dysplasia (Fig. 3c). There was neither dysplasia nor biliary intraepithelial neoplasia on the background mucosa. Immunohistochemical analysis of the mucosal characteristics showed that MUC1, MUC5AC, and MUC6 were positive, whereas MUC2 was negative (Fig. 4). According to the predominant pattern on morphology and the mucin expression form, it was diagnosed as biliary type. Additionally, because Ki67 index was a little less than 10%, it was denied that the tumor was malignant. The postoperative course was good, and the patient was discharged 9 days after the operation. A recurrence has not been detected for 3.5 years.

### Discussion

ICPN was first described as gallbladder lesions of IPNB in the 2010 WHO classification and was classified as premalignant lesions of biliary system in the same category as adenoma, biliary intraepithelial neoplasia, and mucinous cystic neoplasm [2]. IPNB is defined as biliary tumors with an exophytic nature exhibiting papillary mass which can be detected macroscopically within the bile duct lumen, characterized by intraluminal growth [3]. It is associated with mucobilia due to excessive mucin secretion and is more commonly found in the hepatic biliary system [4].

The histopathological characteristics of ICPN are equivalent to IPNB. Four histological subtypes exist, including biliary type, gastric type, intestinal type, and oncocytic type according to IPMN. The mucus glycoprotein expression form is different depending on each histological subtype; that of early biliary carcinoma differs from ICPN [5, 6]. About biliary type, Adsay et al. reported 68% of those represented cancer in situ and 69% included infiltrating cancer [7]. Correlation between mucin expression form and prognosis about several tumors has been studied. The current study showed that MUC1 immunohistochemical staining is a poor prognostic marker for IPNB [5]. Although there are few reports on ICPN, it is considered that this case should be followed closely.

ICPN shows various degrees of dysplasia from low- to high-grade and finally to invasive carcinoma, and the histological findings are often mixed [4]. This variation of dysplastic degree demonstrates the adenoma-carcinoma sequence while papillary adenocarcinoma is assumed to arise through de novo carcinogenesis. Therefore, from the perspective of carcinogenesis, ICPN is distinguished from papillary adenocarcinoma and it is related to the difference of each tumor prognosis. ICPN rarely infiltrates and metastasizes, and the prognosis for ICPN is typically much better than that for gallbladder adenocarcinoma. The 5-year survival rate for ICPN is 60% if including invasive carcinoma and 78% if excluding the invasive region. In contrast, the 5-year survival rate for gallbladder adenocarcinoma is 30% [7]. Therefore, it is important to diagnose ICPN correctly.

However, the concept of ICPN remains unclear about several points.

First, it is difficult to distinguish ICPN, including widespread infiltrating cancerous regions, from gallbladder carcinoma, including papillary tumor regions. It is important to distinguish both for selecting an appropriate treatment strategy. If ICPN is diagnosed preoperatively, simple cholecystectomy can be performed without lymphadenectomy. Second, management for tubular components in ICPN is controversial. There is no clear description about it in the 2010 WHO classification. Adsay et al. defines ICPN as an exophytic intramucosal gallbladder mass greater than 1 cm and composed of dysplastic cells forming a lesion distinct from the neighboring mucosa [7]. According to their definition, the existence of tubular components is not important if the condition mentioned above is satisfied. Additionally, it is considered that macroscopic papillary lesions are not essential for diagnosis.

In the present case, the tumor’s mucosal surface was smooth and its form was similar to a submucosal tumor. Although the macroscopic findings were atypical, histopathological examination showed papillary tumors with low-grade dysplasia and mucin secretion and ICPN was diagnosed. The RAS was formed, the smooth muscle became hyperplastic in the stromal tissue, the tumors arose into the RAS, and the adenomyomatous hyperplasia was merged with ICPN. Thus, the tumor formed like a submucosal tumor.

Although histopathological findings showed papillary tumors with cystic formation, preoperative CT showed only wall thickening and did not show cystic formation findings. It was considered that because the cysts were involved in hyperplastic smooth muscles, the imaging could not detect the cystic formation. On the other hand, the high-intensity punctiform lesions were detected by T2-weighted MRI. It was considered that the finding demonstrated the tumors rise into the RAS, and the cysts were formed.

Although gallbladder adenocarcinoma or ICPN rising in RAS have been reported, to our knowledge, this is the first case of ICPN with adenomyomatous hyperplasia [8]. It is necessary to consider the possibility of tumor lesions within adenomyomatous hyperplasia. As in this case, coexistence of fundal-type adenomyomatous hyperplasia makes diagnosis difficult. In cases of adenomyomatous hyperplasia with irregular wall thickening and sequential changes, it is necessary to follow closely about the merger of the neoplastic lesion.

## Conclusion

We report a case of ICPN that was resembling a submucosal tumor because the tumors rose into the RAS, and adenomyomatous hyperplasia was merged with ICPN. It is necessary to consider the possibility of tumor lesions within adenomyomatous hyperplasia.

## Abbreviations

ICPN: Intracholecystic papillary neoplasm
IPNB: Intraductal papillary neoplasm of the bile duct
RAS: Rokitansky-Aschoff sinus
